# Post-therapeutic circulating tumor cell-associated white blood cell clusters predict poor survival in patients with advanced driver gene-negative non-small cell lung cancer

**DOI:** 10.1186/s12885-023-10985-1

**Published:** 2023-06-22

**Authors:** Ying Wang, Yanxia Liu, Zhiyun Zhang, Baohua Lu, Yuan Gao, Li Tong, Mingming Hu, Peter Ping Lin, Baolan Li, Tongmei Zhang

**Affiliations:** 1 Department of General Medicine, Beijing Chest Hospital, Capital Medical University & Beijing Tuberculosis and Thoracic Tumor Research Institute, Beijing, China; 2 Department of Cancer Research Center, Beijing Chest Hospital, Capital Medical University & Beijing Tuberculosis and Thoracic Tumor Research Institute, Beijing, China; 3 Cytelligen, San Diego, CA USA

**Keywords:** Aneuploid CTCs, CTC-WBC clusters, Treatment response, Disease progression, Survival, Advanced NSCLC

## Abstract

**Purpose:**

This study aimed to investigate the clinical utility of diverse aneuploid circulating tumor cell (CTC) subtypes and particularly CTC-associated white blood cell (CTC-WBC) clusters in predicting treatment response, prognosis and real-time monitoring disease progression in advanced driver gene-negative non-small lung cancer (NSCLC) patients.

**Materials and methods:**

A total of 74 eligible patients were prospectively enrolled and serial blood samples were collected at pre-treatment(t_0_), after two cycles of therapy (t_1_) and at post-four-to-six treatment cycles (t_2_). Co-detection of diverse subtypes of aneuploid CTCs and CTC-WBC clusters was conducted in advanced NSCLC patients receiving first-line treatment.

**Results:**

At baseline, CTCs were detected in 69 (93.24%) patients and CTC-WBC clusters were detected in 23 (31.08%) patients. Patients with CTCs < 5/6ml or with CTC-WBC clusters undetectable exhibited a better treatment response than patients with pre-therapeutic aneuploid CTCs ≥ 5/6ml or harboring CTC-WBC clusters (*p* = 0.034 and *p* = 0.012, respectively). Before treatment, patients bearing tetraploid CTCs ≥ 1/6ml showed significantly inferior progression-free survival (PFS) [hazard ratio (HR):2.420, 95% confidence interval (CI): 1.426–4.106; *p* = 0.001] and overall survival (OS) compared to patients with tetraploid CTCs < 1/6ml (HR:1.907, 95%CI: 1.119–3.251; *p* = 0.018). A longitudinal study demonstrated that post-therapeutic patients harboring CTC-WBC clusters displayed the reduced PFS and OS compared with those without CTC-WBC clusters, and subgroup analysis showed that the presence of CTC-WBC clusters indicated a worse prognosis in both lung adenocarcinoma (LUAD) and lung squamous cell carcinoma (LUSC) patients. After adjusting for multiple significant factors, post-therapeutic CTC-WBC clusters were the only independent predictor of both PFS (HR:2.872, 95% CI: 1.539–5.368; *p* = 0.001) and OS (HR:2.162, 95% CI: 1.168–4.003; *p* = 0.014).

**Conclusions:**

In addition to CTCs, longitudinal detection of CTC-WBC clusters provided a feasible tool to indicate initial treatment response, dynamically monitor disease progression and predict survival in driver gene-negative advanced NSCLC patients.

**Supplementary Information:**

The online version contains supplementary material available at 10.1186/s12885-023-10985-1.

Lung cancer is a devastating disease that is the leading cause of cancer death worldwide [1]. Non-small cell lung cancer (NSCLC) accounts for approximately 85% of all lung cancer cases and more than 60% of patients present locoregional or distant metastases at the time of diagnosis [2]. Although the utilization of small molecule tyrosine kinase inhibitors and immunotherapy in the treatment of NSCLC has led to unprecedented prolonged survival in selected patients [3, 4], the overall prognosis remain disappointing, particularly for those with driver gene-negative advanced disease [5]. According to recent studies, estimated five-year survival rates for driver gene-negative advanced NSCLC vary from 16.3 to 31.9% [6, 7]. Therapeutic resistance and disease progression are the main reasons that contribute to treatment failure and patient death. Hence, there is a need for new biomarkers to better instruct clinical practice in the treatment of advanced NSCLC and a deep understanding of the disease biology is crucial to expand the clinical benefit to a broader patient population.

Circulating tumor cells (CTCs) that detached from the primary tumor or metastases and shed in the patient’s bloodstream can be defined as a category of the functional representatives of tumor status and are regarded as the “seeds” for tumor metastasis. Studies have revealed that CTCs are heterogeneous and exist in the bloodstream as single CTCs, CTC clusters or CTC-white blood cell (WBC) clusters [8]. Despite the rarity in peripheral blood, WBCs can facilitate the seeding process through a direct interaction with CTCs to regulate the cell cycle and CTC-WBC clusters were found to play a crucial role in the promotion of disease progression and tumor metastasis both in circulation and in the tumor microenvironment [9, 10]. Therefore, examination of diverse subtypes of CTCs and CTC-WBC clusters may help to monitor disease progression in real time and better guide the administration of treatment regimens to cancer patients. However, to our knowledge, to date, few reports have focused on the detailed evolutionary characterization of aneuploid CTCs and the clinical significance of CTC-WBC clusters in patients with advanced driver gene-negative NSCLC is not yet fully understood.

Herein, extending beyond our previous work on CTCs and circulating tumor endothelial cells(CTECs) in NSCLC [11, 12], the EpCAM-independent subtraction enrichment and immunostaining-fluorescence in situ hybridization (SE-iFISH) platform was optimized to enrich and perform comprehensive in situ morphologic, karyotypic and phenotypic characterization of a full spectrum of aneuploid CTCs and CTC-WBC clusters in advanced NSCLC receiving first-line treatment. In light of the fact that WBCs possess diverse roles in promoting tumor progression and metastasis, the potential clinical value of CTC-WBC clusters was specifically analyzed with regard to predicting treatment response and timely monitoring of disease progression and survival in driver gene-negative advanced NSCLC.

## Materials and methods

### Patient enrollment and specimen collection

**Fig. 1:**
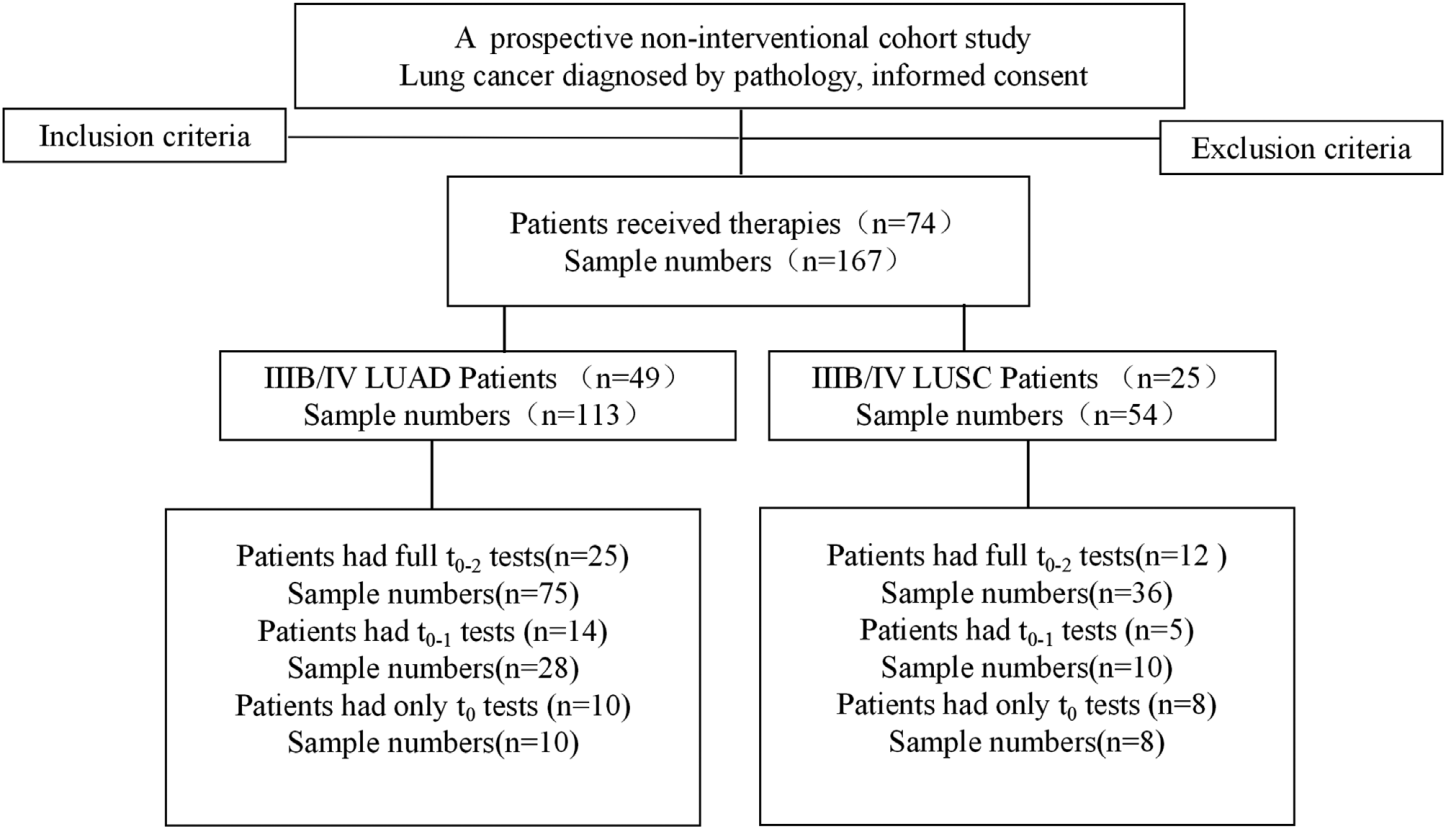
Quantitative illustration of patients and specimens throughout therapy.

This is a prospective, non-interventional, single-center study which aimed to investigate the clinical utility of diverse aneuploid CTCs subtypes and particularly CTC-WBC clusters in advanced NSCLC patients. A total of 74 newly diagnosed patients with inoperable, locally advanced (Stage IIIB) or metastatic (Stage IV) driver gene-negative NSCLC at Beijing Chest Hospital were prospectively enrolled. Among the 74 cases of NSCLC patients, 49 cases of lung adenocarcinoma (LUAD) and 25 cases of lung squamous cell carcinoma (LUSC) were histopathologically diagnosed and genetically validated. Patients who had not received treatment and those who had a performance status (PS) 0–2, with adequate organ function and evaluable tumor lesions were eligible for this study. Patients with a history of other malignant tumors were excluded. Disease stage was determined based on the examination result including computed tomography (CT) of the chest and abdomen, brain magnetic resonance imaging (MRI), radionuclide bone scanning or positron emission tomography (PET) scanning. All participants received the first-line platinum-based chemotherapy alone or plus immunotherapy or anti-angiogenic therapy, concurrent or sequential radiotherapy is preferly administered for those with locally advanced (Stage IIIB) disease except for those intolerable. Six weeks (two cycles) after treatment initiation, evaluation of clinical response was performed by means of standard imaging studies according to Response Evaluation Criteria in Solid Tumors (RECIST) version 1.1 criteria. Responses were recorded as partial response (PR), progressive disease (PD), or stable disease (SD). Maintenance therapy was optional for subjects with PR and SD after the completion of first-line treatment until PD, unacceptable adverse events, or withdrawal from studies. Longitudinal blood samples were collected before treatment administration, after two cycles of chemotherapy and at the time of post-four-to-six treatment cycles, some patients failed to provide blood samples as scheduled due to unforeseeable clinical complications. The primary endpoint was OS and secondary endpoints included PFS and disease control rate (DCR). A total of 167 blood samples were collected from a cohort of 74 patients (see sample collection flow chart in Fig. 1). All participants signed written informed consent before participation in the study.

### Subtraction enrichment of circulating rare cells

Subtraction enrichment was performed according to the manufacturer’s instructions (Cytelligen, San Diego, CA, USA) similar to previously published methods with minor modifications [13]. Briefly, 6 ml of peripheral blood was collected into a tube containing acid citrate dextrose (ACD) anti-coagulant (Becton-Dickinson, Franklin Lakes, NJ, USA). Blood samples were centrifuged at 200×g for 15 min at room temperature to remove plasma. Sedimented blood cells were gently mixed with 3 ml of hCRC buffer, loaded on the non-hematopoietic cell separation matrix in a 50 ml tube, and subsequently centrifuged at 450× g for 5 min. The middle layer containing white blood cells (WBCs) and tumor cells was collected into a 50 ml tube and subsequently incubated with 300 µl of anti-CD45 monoclonal antibody-coated magnetic beads at room temperature for 20 min with gentle shaking. WBCs bound to magnetic beads were depleted using a magnetic separator (Promega, Madison, WI). The bead-free supernatants were collected into a 15 ml tube, followed by adding hCTC buffer to 14 ml. Samples were then spun at 500×g for 4 min at room temperature. Supernatants were aspirated down to 100 µl and resuspended, mixed with the cell fixative (Cytelligen), then applied to formatted slides, and dried for subsequent iFISH analyses.

#### Immunostaining-fluorescence in situ hybridization

With regard to iFISH, dried monolayer cells on the CTC slides were rinsed and incubated with PBS for 3 min, followed by hybridization with centromere probe 8 (CEP8) Spectrum Orange (Vysis, Abbott Laboratories, Chicago, IL, USA). Samples were subsequently incubated with the indicated monoclonal antibodies against cellular proteins including hemocyte biomarker (CD45), endothelial cell biomarker (CD31) and mesenchymal cell biomarker (Vimentin) for 20 min in the dark. After washing, the samples were covered with mounting media containing 4′,6-diamidino-2-phenylindole (DAPI) for nuclear staining (Vector Laboratories, Burlington, CA), and subjected to automated CTC image scanning and analyses. CTCs were identified as DAPI^+^/CD45^−^/CD31^−^/vimentin^+/−^ Chr8 aneuploid cells or DAPI^+^/CD45^−^/CD31^−^/vimentin^+^ Chr8 diploid cells and CTECs were identified as DAPI^+^/CD45^−^/CD31^+^/vimentin^+/−^ Chr8 aneuploid cells or DAPI^+^/CD45^−^/CD31^+^/ vimentin^+^ Chr8 diploid cells, while the CTC-WBC cluster was defined as CTCs adhered to WBCs.

### Statistical analysis

All statistical analyses were conducted using SPSS 25.0 software (Chicago, IL, USA). Chi-square tests and Fisher’s exact tests were applied to compare categorical data. Continuous data were expressed as the median and interquartile range (IQR) where appropriate. The Mann-Whitney U-test was applied to compare continuous variables between two groups. Kaplan-Meier survival plots for PFS and OS were created based on diverse aneuploid CTCs or CTC-WBC clusters and survival curves were compared using log-rank or Breslow tests. Uni-variate and multi-variate Cox proportional hazards regression models with HRs and 95% Cis were applied to determine the independent prognostic factors for PFS and OS. All p values were two-sided. P < 0.05 was considered statistically significant.

## Results

### Baseline aneuploid CTCs and CTC-WBC clusters correlated with treatment response in advanced NSCLC patients

From January 2018 to July 2022, a total of 74 advanced driver gene-negative NSCLC patients were enrolled in this study. Patients with advanced-stage cancer included 49 cases of LUAD and 25 cases of LUSC, and the clinical characteristics of the included patients are listed in Table 1. As of the cutoff date of October 30, 2022, 57 patients died and the median OS was 19.415 months (range, 1.13 to 55.77 months). Among the cohort of 74 subjects recruited in this follow-up clinical study, 18 participants failed to provide serial blood samples and a total of 167 blood samples that covered baseline (t_0_) and post-treatment blood draws (t_1_ and t_2_) were collected (Fig. 1).

**Table 1:** Clinical characteristics of enrolled NSCLC patients

Characteristics	Number of patients (%)	Characteristics	Number of patients (%)
Age (year)		Liver metastasis	
<60	22(29.73%)	Yes	6(8.11%)
≥60	52(70.27%)	No	68(91.89%)
Sex		Bone metastasis	
Male	60(81.08%)	Yes	22(29.73%)
Female	14(18.92%)	No	52(70.27%)
Smoking history		Brain metastasis	
Yes	58(78.38%)	Yes	4(5.41%)
No	16(21.62%)	No	70(94.59%)
Histology		Efficacy	
Adenocarcinoma	49(66.22%)	PR	16(21.62%)
Squamous	25(33.78%)	SD	48(64.86%)
PS score		PD	10(13.52%)
0	3(4.05%)	Treatment regimen	
1	71(95.95%)	Bevacizumab + Chemotherapy	45(60.81%)
TNM stage		Immunotherapy + Chemotherapy	21(28.38%)
IIIB	12(16.22%)	Immunotherapy	8(10.81%)
IV	62(83.78%)		

**Fig. 2:**
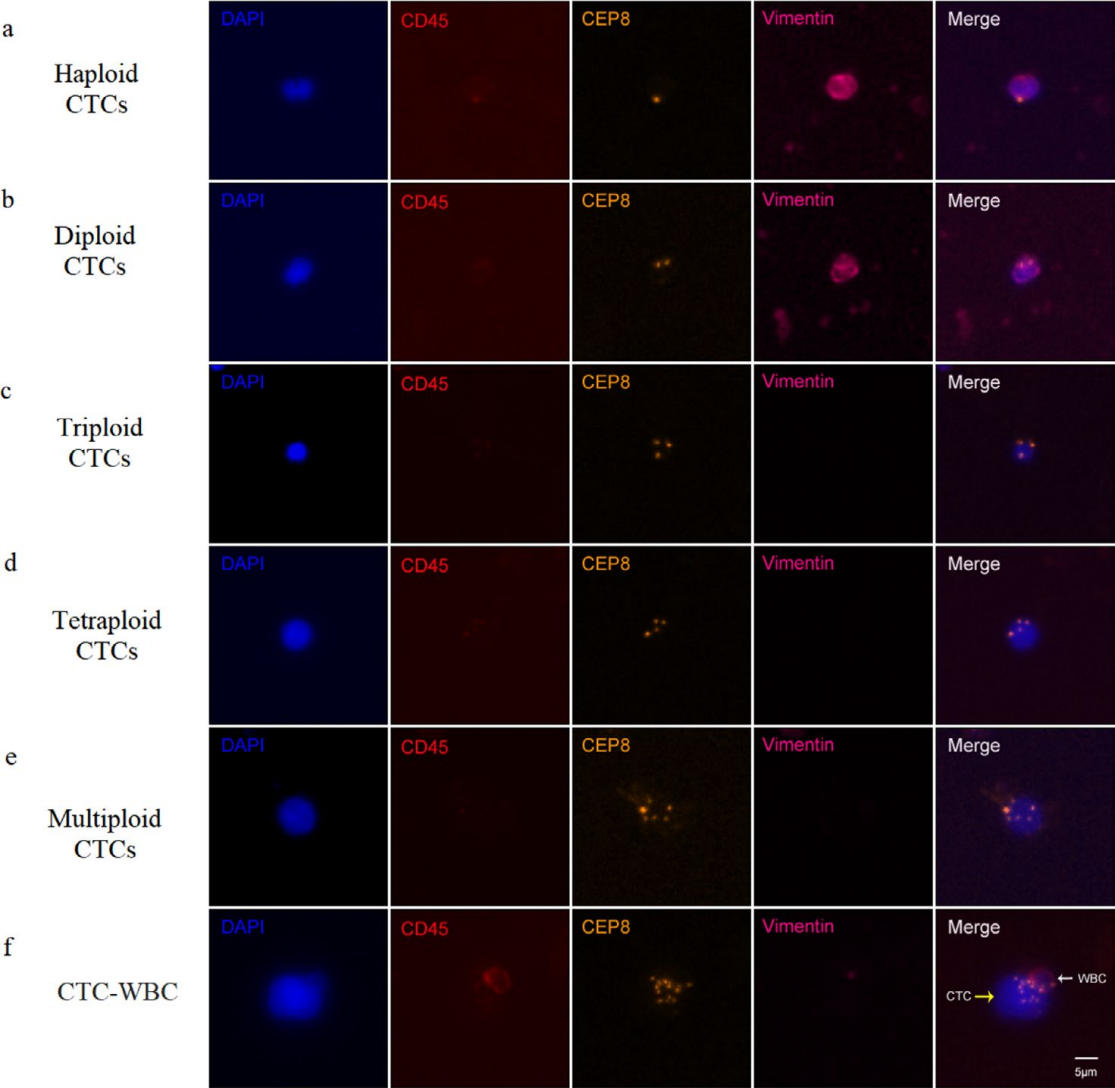
Representative images of CTC subtypes and CTC-WBC identifified by iFISH. **A**. A representative image of a haploid CTCs (DAPI+/CD45-/Vimentin + cells with haploid Chr8). **B**. A representative image of a diploid CTCs (DAPI+/CD45-/Vimentin + cells with diploid Chr8). **C**. A representative image of a triploid CTCs (DAPI+/CD45-/Vimentin- cells with triploid Chr8). **D**. A representative image of a tetraploid CTCs (DAPI+/CD45-/Vimentin- cells with tetraploid Chr8). **E**. A representative image of a multiploid CTCs (DAPI+/CD45-/Vimentin- cells with multiploid Chr8). **F**. A representative image of a CTC-WBC cluster (the yellow arrow indicates CTC, and the white arrow indicate WBC adhered to a CTC)

Six-channel SE-iFISH was applied to characterize CTCs based on cell size, aneuploidy of chromosome 8 (Chr8) and phenotype expression. Representative images of CTCs with different morphological and aneuploidy Chr8 types along with CTC-WBC clusters are shown in Fig. 2. Before treatment, CTCs were detected in 69 out of 74 (93.24%) patients and the positivity rate of CTC-WBC clusters was 31.08% (23/74). The number of CTCs and CTC-WBC clusters in each patient ranged from 0-296/6ml and 0–25/6ml, respectively. The correlation of aneuploid CTCs and CTC-WBC clusters in pretreatment patients with clinical characteristics was investigated. The obtained results revealed that LUAD patients tended to have a higher CTC-WBC clusters than LUSC patients (*p* = 0.045, Suppl Table 1), whereas age, sex, smoking history and performance status (PS) were not found to be significantly associated with CTCs or CTC-WBC clusters.

**Fig. 3:**
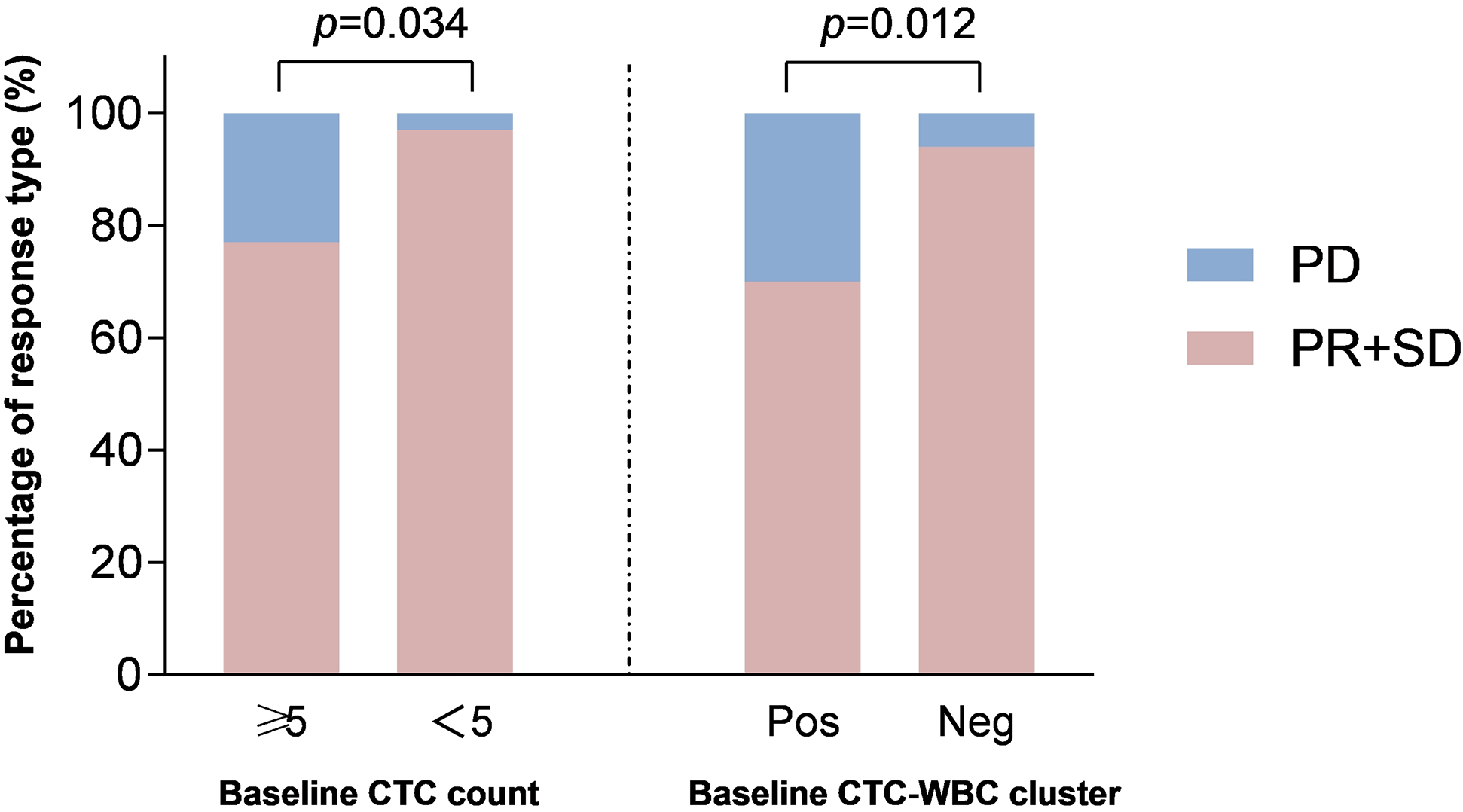
The relationship between CTCs, CTC-WBC clusters and disease control rate in NSCLC.

Early identification of non-responders to initial treatment is of great significance to better instruct clinical practice in cancer management and CTCs subtypes as well as CTC-WBC clusters may provide potential circulating biomarkers related to treatment resistance. Therefore, we evaluated the relationship between CTCs, CTC-WBC clusters and treatment efficiency. The results revealed that patients with CTC-CTCs ≥ 5/6 ml, CTC-WBC clusters ≥ 1/6 ml were significantly correlated with a decreased disease control rate (*p* = 0.034 and *p* = 0.012, respectively, Fig. 3). Our results suggest that in addition to CTC enumeration, the baseline presence of CTC-WBC clusters may also serve as biomarkers for the initial treatment response in advanced NSCLC patients.

### Categorical analysis of aneuploid CTCs in advanced lung cancer patients

**Fig. 4:**
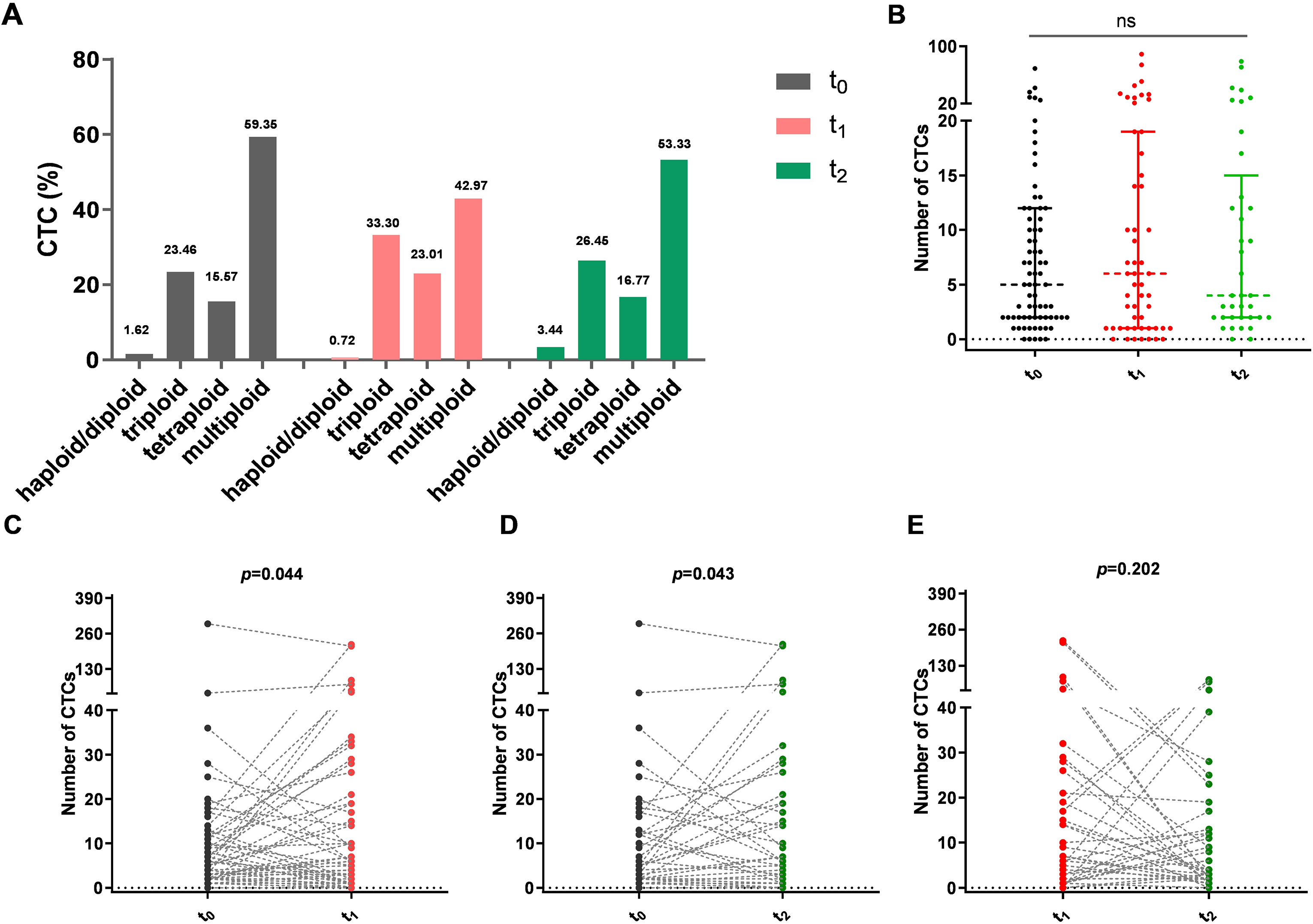
Analysis of aneuploid CTCs at baseline and after treatment. (**A**). CTCs compositional waterfall map of different karyotypic characterization. (**B**). Distribution of CTCs in all blood samples at baseline (t_0_) and post-treatment (t_1_ and t_2_). (**C**). CTCs variation trend of paired blood samples at t_0_ and t_1_ time points. (**D**). CTCs variation trend of paired blood samples at t_0_ and t_2_ time points. (**E**). CTCs variation trend of paired blood samples at t_1_ and t_2_ time points

CTCs provide an optimal way to better understand the heterogeneity and the evolutionary changes in tumor cells along with treatment administration in advanced lung cancer patients. Compositional analysis of CTCs subtypes at different time points in 74 patients was investigated, and the obtained results showed that pretreatment multiploid CTCs constituted the principal karyotype of all CTCs with a proportion of 59.35%, while the second to the largest of CTCs subpopulations were triploid CTCs followed by tetraploid CTCs and haploid/diploid CTCs which accounted for 23.46%, 15.57% and 1.62% of the baseline CTC number (Fig. 4A). Further karyotype variations of CTCs showed similar proportions of Chr8 haploid/diploid, triploid, tetraploid, and multiploidy cells over time(t_0 − 2_) (Fig. 4A). Numerical analysis of aneuploid CTCs at three time points in all blood samples was conducted but the difference did not reach statistical significance in this study (*p* = 0.905, Mann-Whitney U-test) (Fig. 4B).

For 37 patients who had a full t_0 − 2_ test, a paired Mann-Whitney U-test was used to analyze cell number variation. The median numbers of CTCs were 6 (t_0_, blue, IQR 2-16.5), 5 (t_1_, purple, IQR 2–12), and 4 (t_2_, purple, IQR 2–15). The quantity of CTCs after treatment significantly decreased compared to that of pre-treatment (t_0_vs. t_1_, *p* = 0.044; t_0_vs. t_2_, *p* = 0.043, Fig. 4C and D), however, the difference between the values of t_1_ and t_2_ did not reach statistical significance in this study (*p* = 0.202, Fig. 4E).

### Pretreatment tetraploid CTCs, triploid CTCs and CTC-WBC clusters predict poor prognosis in advanced NSCLC patients

**Fig. 5:**
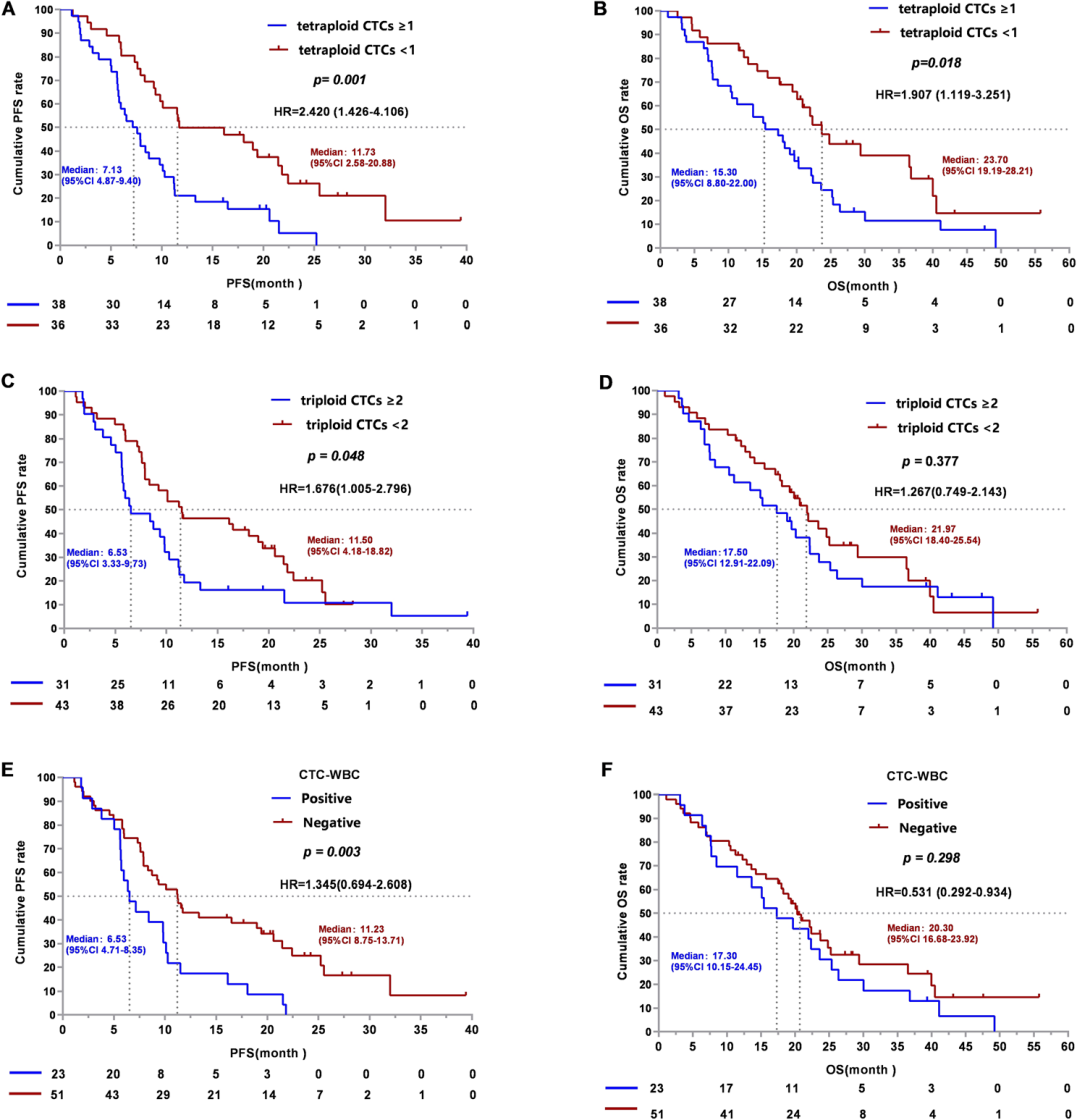
Analysis of baseline CTC counts and CTC-WBC clusters with NSCLC patients’ survival. (**A**). The PFS survival curves of NSCLC patients with baseline tetraploid CTCs ≥ 1 and tetraploid CTCs < 1. (**B**). The OS survival curves of NSCLC patients with baseline tetraploid CTCs ≥ 1 and tetraploid CTCs < 1. (**C**). The PFS survival curves of NSCLC patients with baseline triploid CTCs ≥ 2 and triploid CTCs < 2. (**D**). The OS survival curves of NSCLC patients with baseline triploid CTCs ≥ 2 and triploid CTCs < 2. (**E**). The PFS survival curves of NSCLC patients with negative and positive baseline CTC-WBC clusters. (**F**). The OS survival curves of NSCLC patients with negative and positive baseline CTC-WBC clusters

It is well accepted that CTCs levels are effective in predicting the survival of lung cancer [14]. However, the prognostic value of diverse aneuploid CTC subtypes in NSCLC is rarely reported. Analysis of CTCs based on Chr8 aneuploidy and the correlation of aneuploidy CTCs with PFS and OS were conducted in our study. Patients harboring tetraploid CTCs ≥ 1/6ml showed a shorter median PFS of 7.13 months (95% CI: 4.87–9.40 months) compared to 11.73 months (95% CI: 2.58–20.88 months) for patients with tetraploid CTCs < 1/6ml. The difference in PFS was statistically significant (*p* = 0.001, log-rank test, Fig. 5A). Further analysis was performed to examine the relationship between tetraploid CTCs and OS, and the results revealed that patients possessing tetraploid CTCs ≥ 1/6ml had a median OS of 15.4 months (95% CI: 8.80–22.00 months), which was shorter than 23.7 months (95% CI: 19.19–28.21 months) for patients who had no tetraploid CTCs detected, and the difference between the values of OS was statistically significant (*p* = 0.018, log-rank test, Fig. 5B), indicating that the presence of tetraploid CTCs had relevance to patients’ shorter PFS and OS. Similar results were observed with respect to the association between triploid CTCs and PFS (*p* = 0.048, log-rank test, Fig. 5C) but not OS (*p* = 0.377, log-rank test, Fig. 5D).

To further assess the relationship between CTC-WBC clusters and patient prognosis, patients were stratified into two groups according to the detection status of the CTC-WBC clusters. Compared to patients without CTC-WBC clusters, patients with CTC-WBC clusters showed significantly shorter PFS [6.53 (95% CI, 4.71–8.35) months vs. 11.23 (95% CI, 8.75–13.71) months, *p* = 0.003 log-rank test, Fig. 5E]. However, no statistical significance was achieved in OS between the two groups in our study [20.30 (95% CI, 16.68–23.92) months vs. 17.30 (95% CI, 10.15–24.45) months, *p* = 0.298 log-rank test, Fig. 5F].

### Post-therapeutic CTC-WBC clusters independently predict PFS and OS in NSCLC

Of the total cohort of 74 enrolled subjects, 56 were available for at least 2 blood samples. Among them, 7 had consistent CTC-WBC clusters, and 10 acquired post-therapeutic CTC-WBC clusters during therapy. Meanwhile, 29 patients showed a nonchanged stable CTC-WBC cluster absence status and 10 had CTC-WBC clusters eliminated following chemotherapy.

**Fig. 6:**
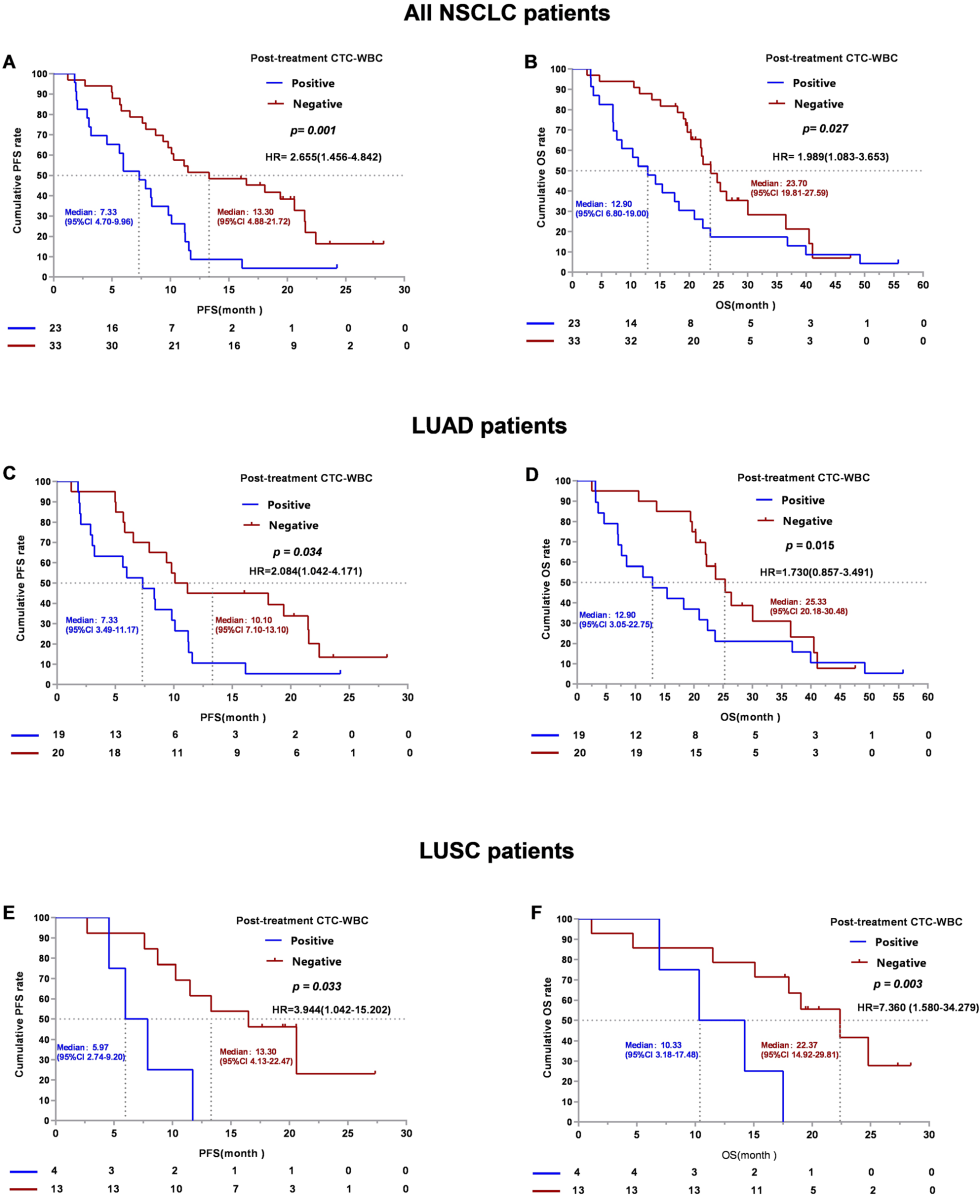
Analysis of post-therapeutic CTC-WBC clusters with NSCLC patients’ survival. (**A**). The PFS survival curves of NSCLC patients with negative and positive post-therapeutic CTC-WBC clusters. (**B**). The OS survival curves of NSCLC patients with negative and positive post-therapeutic CTC-WBC clusters. (**C**). The PFS survival curves of LUAD patients with negative and positive post-therapeutic CTC-WBC clusters. (**D**). The OS survival curves of LUAD patients with negative and positive post-therapeutic CTC-WBC clusters. (**E**). The PFS survival curves of LUSC patients with negative and positive post-therapeutic CTC-WBC clusters. (**F**). The OS survival curves of LUSC patients with negative and positive post-therapeutic CTC-WBC clusters

The presence of post-therapeutic CTC-WBC clusters (t_1 − 2_) demonstrated a significantly shorter median PFS of 7.33 months (95% CI:4.70–9.96 months) compared to a median PFS of 13.30 months (95% CI: 4.88–21.72 months) for those with the absence of CTC-WBC clusters (t_1 − 2_) (*p* = 0.001, log-rank test, Fig. 6A). Similar results were observed with respect to the relationship between post-therapeutic CTC-WBC clusters and OS. Patients with post-therapeutic CTC-WBC clusters (t_1 − 2_) had significantly reduced median OS compared to those without CTC-WBC clusters (t_1 − 2_) [12.90 (95% CI, 6.80–19.00) months vs. 23.70 (95% CI, 19.81–27.59) months, *p* = 0.027, log-rank test, Fig. 6B]. To fully investigate the prognostic value of CTC-WBC clusters in different pathological types, subgroup analysis was conducted as well. The obtained data demonstrated that the post-therapeutic presence of CTC-WBC clusters indicated inferior PFS and OS in both the LUAD (*p* = 0.034 and *p* = 0.015 respectively, Fig. 6C and D) and LUSC cohorts (*p* = 0.033 and *p* = 0.003 respectively, Fig. 6E and F).

We then analyzed the impact of baseline variables including age, sex, disease stage, smoking history and PS score, on PFS and OS. Variables that were of statistical significance in univariate analysis were included in a multivariate Cox proportional hazard model. Finally, CTC-WBC clusters emerged as independent prognostic factors associated with PFS (*p* = 0.001, Table 2) and OS (*p* = 0.014, Table 3). The obtained results further validated that CTC-WBC clusters detected after treatment are an independent prognostic factor for both PFS and OS in advanced driver gene-negative NSCLC patients.

**Table 2:** Univariate and multivariate analysis for PFS predictors in advanced NSCLC patients

Variables	Univariate Analysis		Multivariate Analysis	
	HR(95%CI)	*p*	HR(95%CI)	*p*
Age ≥ 60 vs. < 60	1.123(0.612–2.060)	0.708		
Gender male vs. female	0.858(0.427–1.721)	0.666		
PS 1–2 vs. 0	0.535(0.072–3.976)	0.541		
Smoking history Yes vs. No	0.598(0.306–1.168)	0.132		
Pathological type LUAD vs. LUSC	1.266(0.734–2.185)	0.396		
TNM stage III vs. VI	1.112(0.576–2.147)	0.752		
Baseline triploid CTCs ≥ 2 vs. < 2	1.676(1.005–2.796)	0.048	1.682(0.858–3.298)	0.130
Baseline tetraploid CTCs ≥ 1 vs. < 1	2.420(1.426–4.106)	0.001	1.866(0.953–3.655)	0.069
Baseline CTC-WBC Clusters Pos vs. < Neg	2.238(1.312–3.819)	0.003	1.345(0.694–2.608)	0.380
Post-treatment CTC-WBC Clusters Pos vs. < Neg	2.655(1.456–4.842)	0.001	2.872(1.539–5.368)	0.001

**Table 3:** Univariate and multivariate analysis for OS predictors in advanced NSCLC patients

Variables	Univariate Analysis		Multivariate Analysis	
	HR(95%CI)	*p*	HR(95%CI)	*p*
Age ≥ 60 vs. < 60	1.343(0.748–2.413)	0.324		
Gender male vs. female	0.651(0.341–1.242)	0.193		
PS 1–2 vs. 0	0.666(0.091–4.864)	0.688		
Smoking history Yes vs. No	0.692(0.369–1.299)	0.252		
Pathological type LUAD vs. LUSC	0.764(0.422–1.382)	0.373		
TNM stage III vs. VI	1.097(0.535–2.250)	0.800		
Baseline triploid CTCs ≥ 2 vs. < 2	1.267(0.749–2.143)	0.377		
Baseline tetraploid CTCs ≥ 1 vs. < 1	1.907(1.119–3.251)	0.018	1.669(0.904–3.082)	0.102
Baseline CTC-WBC Clusters Pos vs. < Neg	1.329(0.778–2.270)	0.298		
Post-treatment CTC-WBC Clusters Pos vs. < Neg	1.989(1.083–3.653)	0.027	2.162(1.168–4.003)	0.014

## Discussion

This study provided evidences for noninvasive tumor monitoring during treatment, revealing the clinical value of heterogeneous aneuploid CTCs and that longitudinal detection of CTC-WBC clusters had the potential for the indication of tumor response, prediction of disease progression and survival outcomes in driver gene-negative NSCLC patients receiving first-line therapy.

Although biomarkers such as PD-L1 can serve as predictive indicators interrogated with immune-checkpoint inhibitors and provide hints for treatment decisions [15], identifying high-risk advanced driver gene-negative NSCLC patients to improve clinical outcomes has remained a challenge until now. CTC detection is an indisputable cornerstone of the liquid biopsy method allowing for tumor screening, disease monitoring and patient prognosis prediction in lung cancer patients [16, 17]. Although the development of CTCs has accelerated the understanding of tumor biology to a large extent, no consensus has been reached on the platform to detect and isolate CTCs and the heterogeneity of diverse subtype of CTCs remains to be investigated. In most of the current assays for the detection of CTCs, the platforms are mainly based on the cell size and expression of epithelial markers such as EPCAM and cytokeratins [18]. However, evolutionary changes in anchor proteins and cell size inevitably result in a considerable amount of CTCs being undetectable. Here, in our study, a new CTC isolation platform SE-iFISH, was applied to enrich CTCs with high efficiency and revealed an important CTC heterogeneity based on cell size and karyotyping of Chr8 ploidy in advanced NSCLC patients. The obtained results revealed a relatively higher CTC-positive rate of 93.24% than the detection sensitivity demonstrated by traditional technologies that rely on EpCAM expression [19]. As is known, CTCs commonly circulate as single cells but can also occasionally found to form clusters. The positivity of CTC-WBC clusters in our study was 31.08% which is in accordance with published observations and much higher than 9.52% in NSCLC patients with early stages [20]. Further correlation analysis showed that the presence of CTC-WBC clusters was significantly associated with pathology type, and CTC-WBC cluster events were more likely to occur in advanced stages of LUAD patients than in those with LUSC.

Previous studies performed by others indicated that the amount of CTCs fluctuated during the therapeutic process [21]. It has been proven that CTCs are highest at the time of diagnosis and disease progression and are suppressed while on treatment [22], and the number of total CTCs is significantly decreased after chemotherapy [23]. Here, aside from analysis of total CTCs number variation at each time point in all 167 blood samples, extensive investigation regarding to CTC variation trends was conducted in patients who had a full t_0 − 2_ test. Similarly, in agreement with the above reports, the present study also demonstrated that the median values of post-treatment CTCs (t_1_ or t_2_) were statistically reduced compared with CTCs at baseline, whereas no significant difference in enumeration between t_1_ and t_2_ was observed in our data.

The clinical utility of CTCs has been validated by a variety of investigations, however, to our knowledge, few studies have been conducted to systemically investigate how diverse CTCs based on Chr8 aneuploidy correlate with clinical outcome in NSCLC. The prognostic significance of different subtypes of aneuploid CTCs was evaluated in the current study. Based on karyotyping of Chr8 ploidy, CTCs were subcategorized as haploid, diploid, triploid, tetraploid and multiploidy CTCs in advanced NSCLC patients. For the quantitative composition, multiploid CTCs accounted for the majority of the absolute CTC number followed by triploid CTCs, tetraploid CTCs and haploid/diploid CTCs in pretreatment patients. Although triploid and tetraploid CTCs accounted for no more than half of the entire CTC number, the obtained results showed that baseline tetraploid CTCs were significantly correlated with decreased PFS and OS in advanced NSCLC patients. Meanwhile, pretreatment triploid CTCs were proven to be associate with worsened PFS, which was in line with our previous reports [24]but discordant with the conclusion that triploid CTCs are of no clinical significance in prognosis in gastric cancer [25]. The discrepancy in the above studies may be due to the biological differences in various cancer types. Taken together, detailed karyotype characterization is of great importance in better understanding the heterogeneity of tumor cells and the specific subpopulations of tetraploid and triploid CTCs are key indicators in predicting poor prognosis in advanced driver gene-negative NSCLC patients.

Tumor cells are highly heterogeneous and growing evidence demonstrates that different cancer cell clones can show cooperative behavior, promoting mutual survival and metastatic ability [26]. In addition to cancer cell-cancer cell interactions, cancer cell-immune cell communication plays a crucial role in the process of disease progression and metastasis colonization [27] and the interaction between CTCs and neutrophils is essential [28, 29]. It has been proven that WBC can accelerate the seeding process through a direct interaction with CTCs [9] and CTC-WBC clusters have recently attracted increasing attention because of their contribution to drug resistance and cancer-promoting effects [8]. Although the clinical utility of CTCs subtypes and CTC-WBC clusters for evaluation of tumor response, prediction of disease progression and survival outcomes have been explored by an increasing number of studies [10], the role of CTC-WBC cluster in the evaluation of tumor response, prediction of disease progression and survival outcomes in advanced NSCLC remain largely uncharacterized. To the best of our knowledge, only one study has explored the clinical value of CTC-WBC clusters in NSCLC. Nevertheless, the study mainly focused on the correlation between CTC-WBC clusters and disease progression. Whether CTC-WBC clusters can be used to indicate initial treatment response or predict PFS and OS in driver gene-negative advanced NSCLC patients is still unclear. The present study suggested that the baseline presence of CTC-WBC clusters is significantly correlated with decreased DCR and that positive detection of CTC-WBC clusters might possess clinical utility in predicting NSCLC patients’ drug resistance to initial treatment. Moreover, survival analysis of the pre-treatment patients confirmed that baseline CTC-WBC cluster-negative patients exhibited significantly longer PFS than those with CTC-WBC cluster-positive detection, which was in line with the previous research conducted by Li [20]. However, baseline CTC-WBC clusters can not predict OS according to our data. Since longitudinal detection of CTC-WBC clusters is available in the majority of enrolled patients, we, therefore, evaluated the prognostic significance of post-therapeutic CTC-WBC clusters in the current study. The obtained results showed that the presence of post-therapeutic CTC-WBC clusters independently predicted poor PFS and OS in patients with advanced NSCLC. Similar findings have been proven in advanced renal cell carcinoma [10], liver cancer [30], breast cancer [31], and gastric cancer [32]. Our data highlight the prognostic performance of CTC-WBC clusters in NSCLC and longitudinal detection of CTC-WBC clusters may help clinicians better assess patient outcomes and develop more personalized treatment strategies. A previous study has investigated the gene profiles of cells in CTC-WBC clusters by single-cell RNA sequencing, and the results revealed that the majority of CTCs were associated with neutrophils. A number of genes that regulate the cell cycle were established differentially expressed in neutrophils adhering to CTCs compared to CTCs alone, thus promoting to more efficient metastasis formation [9]. Moreover neutrophils could bind to CTCs through intercellular adhesion molecule-1 to protect CTCs from the attack by natural killer cells or macrophages and facilitate extravasation [33]. Therefore, the underlying mechanisms of CTC-WBC clusters in promoting disease progression might be related to the enhanced cell metastasis formation and immune escape of WBCs combined with CTCs. However, further investigations are needed to better understand cancer cell-immune cell communication mechanisms.

In conclusion, our findings indicated that CTC-WBC clusters could not only monitor disease progression during or after first-line treatment but are also a promising noninvasive biomarker for indicating initial treatment response and predicting survival in NSCLC patients. Longitudinal detection of CTC-WBC clusters may have implications in the risk stratification of advanced driver gene-negative NSCLC patients for personalized clinical management.

Our study has several limitations that should be noted. First, this single center study involved a relatively small sample size, which may limit the statistical power of the analysis. Second, the data were based on two cohorts (LUAD cohort and LUSC cohort) study according to pathological type which may make the conclusions less comprehensive. Finally, the underlying mechanism of CTC-WBC clusters in treatment resistance and disease progression remains unclear and needs to be further elucidated. 

## Electronic supplementary material

Below is the link to the electronic supplementary material.

**Supplemental Table 1**: Correlation analysis between CTCs/CTC-WBC clusters and the clinical characteristics of advanced NSCLC patients

## Data Availability

All datasets are available in the supplementary materials or upon request from the corresponding author for reasonable request.

## Abbreviations

CEP8: Centromere probe 8
Chr8: Chromosome8
CT: Computed tomography
CTCs: Circulating tumor cells
CTC-WBC clusters: Circulating tumor cell-associated white blood cell clusters
CTECs: Circulating tumor endothelial cells
DCR: Disease control rate
HR: Hazard ratio
IQR: Interquartile range
LUAD: Lung adenocarcinoma
LUSC: Lung squamous cell carcinoma
MRI: Magnetic resonance imaging
NSCLC: Non-small cell lung cancer
OS: Overall survival
PB: Peripheral blood
PD: Progressive disease
PET: Positron emission tomography
PFS: progression-free survival
PR: Partial response
RECIST: Response Evaluation Criteria in Solid Tumors
SD: Stable disease
SE-iFISH: Subtraction enrichment and immunostaining-fluorescence in situ hybridization
