# Supplementary material for: Post-therapeutic circulating tumor cell-associated white blood cell clusters predict poor survival in patients with advanced driver gene-negative non-small cell lung cancer
Source: BMC Cancer. 2023 Jun 22;23:578. doi: 10.1186/s12885-023-10985-1 (PMC10286386; doi:10.1186/s12885-023-10985-1)
Supplement: Supplementary file 1 — Supplemental Table 1: Correlation analysis between CTCs/CTC-WBC clusters and the clinical characteristics of advanced NSCLC patients [file 12885_2023_10985_MOESM1_ESM.pdf]

Supplemental Table 1 Correlation analysis between CTCs/CTC-WBC clusters and the clinical characteristics of advanced NSCLC patients

Characteristics	Number of patients (%)	≥5/6ml CTC count (%)	*p*	≥1/6ml CTC-WBC cluster (%)	*p*
Age (year)			0.649		0.929
<60	22(29.73%)	11(14.86%)		7(9.46%)	
≥60	52(70.27%)	29(39.19%)		16(21.62%)	
Sex			0.351		0.235
Male	60(81.08%)	34(45.95%)		21(28.38%)	
Female	14(18.92%)	6(8.11%)		2(2.70%)	
Smoking history			0.842		0.369
Yes	58(78.38%)	31(41.89%)		20(27.03%)	
No	16(21.62%)	9(12.16%)		3(4.05%)	
Histology			0.083		0.045
Adenocarcinoma	49(66.22%)	30(40.54%)		19(25.68%)	
Squamous	25(33.78%)	10(13.51%)		4(5.41%)	
PS score			0.886		0.679
0	3(4.05%)	1(1.35%)		1(1.35%)	
1-2	71(95.95%)	39(52.70%)		22(29.73%)	
TNM stage			0.347		0.402
IIIB	12(16.22%)	5(6.76%)		2(2.70%)	
IV	62(83.78%)	35(47.30%)		21(28.38%)	
Liver metastasis			0.525		0.559
Yes	6(8.11%)	2(2.70%)		3(4.05%)	
No	68(91.89%)	38(51.35%)		20(27.03%)	
Bone metastasis			0.649		0.523
Yes	22(29.73%)	11(14.86%)		8(10.81%)	
No	52(70.27%)	29(39.19%)		15(20.27%)	
Brain metastasis			0.727		0.409
Yes	4(5.41%)	3(4.05%)		0(0.00%)	
No	70(94.59%)	37(50.00%)		23(31.08%)	
